# Should all paediatric patients with presumed idiopathic scoliosis undergo MRI screening for neuro-axial disease?

**DOI:** 10.1007/s00381-018-3878-7

**Published:** 2018-07-27

**Authors:** Patrick A. Tully, Ben A. Edwards, Omar Mograby, Harriet S. M. Davis, Oluwole Arieskola, Shailendra Magdum, Prashanth Rao, Jayaratnam Jayamohan

**Affiliations:** 1 Department of Paediatric Neurosurgery, John Radcliffe Hospital, Oxford University Hospitals NHS Foundation Trust, Headley Way, Headington, Oxford, OX3 9DU UK; 2 Nuffield Department of Clinical Neurosciences, University of Oxford, Level 6, West Wing, John Radcliffe Hospital, Oxford, OX3 0DU UK; 3 Nuffield Department of Surgical Sciences, University of Oxford, John Radcliffe Hospital, Headington, Oxford, OX3 9DU UK; 4 Department of Neurosurgery, Westmead Hospital, Western Sydney Local Health District, Westmead, NSW 2145 Australia; 5 Nuffield Orthopaedic Centre, Oxford University Hospitals NSH Foundation Trust, Oxford, UK

**Keywords:** Idiopathic scoliosis, Chiari malformation, Surgery, Foramen magnum decompression

## Abstract

**Background:**

Idiopathic scoliosis is a relatively common childhood condition affecting 0.47–5.2% of the population. Traditional interventions focus on orthopaedic correction of the curve angle. There is a spectrum of patients with scoliosis who are found to have neuro-axial abnormality on full MRI of the spine, but not all surgeons request imaging in the absence of neurological symptoms. There is evidence to suggest that treatment of neuro-axial disease may improve scoliosis curve outcome. We therefore sought to estimate what proportion of patients with normal neurology and scoliosis are found to have neuro-axial abnormality on full MRI imaging of the spine, in particular Chiari malformation and syringomyelia.

**Results:**

Out of 11 identified studies consisting of 3372 paediatric patients (age < 18 years), mean weighted proportion demonstrates that 14.7% of patients with scoliosis (Cobb angle > 20°) and normal neurological examination will demonstrate a neuro-axial abnormality on full MRI imaging of the spine. Of patients, 8.3 and 8.4% were found to have Chiari malformation and syringomyelia, respectively.

**Conclusions:**

Up to one in seven paediatric patients with scoliosis and normal neurological examination will demonstrate neuro-axial disease on MRI imaging of the spine. Given that younger age and earlier age of decompression is associated with improvement in curve angle, it seems important that MRI screening be considered in all patients regardless of neurological examination findings. There is a potentially long-term benefit in these patients. Multi-cross institutional prospective studies are encouraged to further investigate effect on curve angle.

## Introduction

Idiopathic scoliosis has an overall prevalence of 0.47–5.2% [1]. There is evidence to suggest an association between scoliosis and neuro-axial disease (NAD) [2]. Chiari malformation (CM) and/or syringomyelia are the most common neuro-axial abnormalities associated with scoliosis [3–5]. Foramen magnum decompression (FMD) in the context of CM and syringomyelia has been shown to improve scoliosis curve and reduce progression, some suggesting ages less than 10 benefit the most [4, 6–8]. It is hypothesised that the pathology can result in denervation/irregular contraction of the deep back muscles, therefore removing such a neurologic driver aids reduction in progression in scoliosis curve [6, 9]. There is a spectrum of patients with idiopathic scoliosis who are found to have a neurological abnormality on imaging, but not all surgeons request a spine MRI for scoliosis in the absence of focal neurological deficit. This poses a potential intervention point if identifying such disease may improve long-term quality of life and scoliosis curve [3, 6, 9–11]. We therefore sought to estimate what proportion of patients with normal neurology and scoliosis are found to have neuro-axial abnormality on full MRI imaging of the spine. This is a question that has not been previously investigated.

## Aims

This study aims to determine the proportion of paediatric patients diagnosed with idiopathic scoliosis and normal neurological examination who are found to have a neuro-axial abnormality on full spine MR imaging, in particular the proportion of patients diagnosed with CM malformation and syringomyelia.

## Methods

We searched PubMed (National Library of Medicine, http://www.ncbi.nlm.nih.gov), EMBASE (Elsevier, http://www.elsevier.com/online-tools/embase) and MEDLINE (Pro Quest, http://search.proquest.com/medline). The PubMed, EMBASE and MEDLINE databases search was combined on NICE HDAS (Healthcare databases advanced search, https://www.hdas.nice.org.uk). The results were then copied to Endnote (Thomas Reuters, http://www.endnote.com), and duplications were removed. The searches included all dates up to April 2017 using the following search terms: Chiari Malformation AND Scoliosis. For the purposes of this study, a paediatric population defined as less than 18 years of age. The inclusion criteria include patients with documented idiopathic scoliosis with a Cobb angle > 20 or greater, normal neurological examination and a full brain and spine MRI. Exclusion criteria included patients > 18 years of age, scoliosis diagnosed with Cobb angle < 20°, primary condition investigated not idiopathic scoliosis, abnormal neurological examination, and studies that retrospectively reviewed patient with diagnosed neuro-axial disease to determine the association with scoliosis. We excluded case reports, letters, comments, reviews and non-English language studies.

## Results

**Fig. 1:**
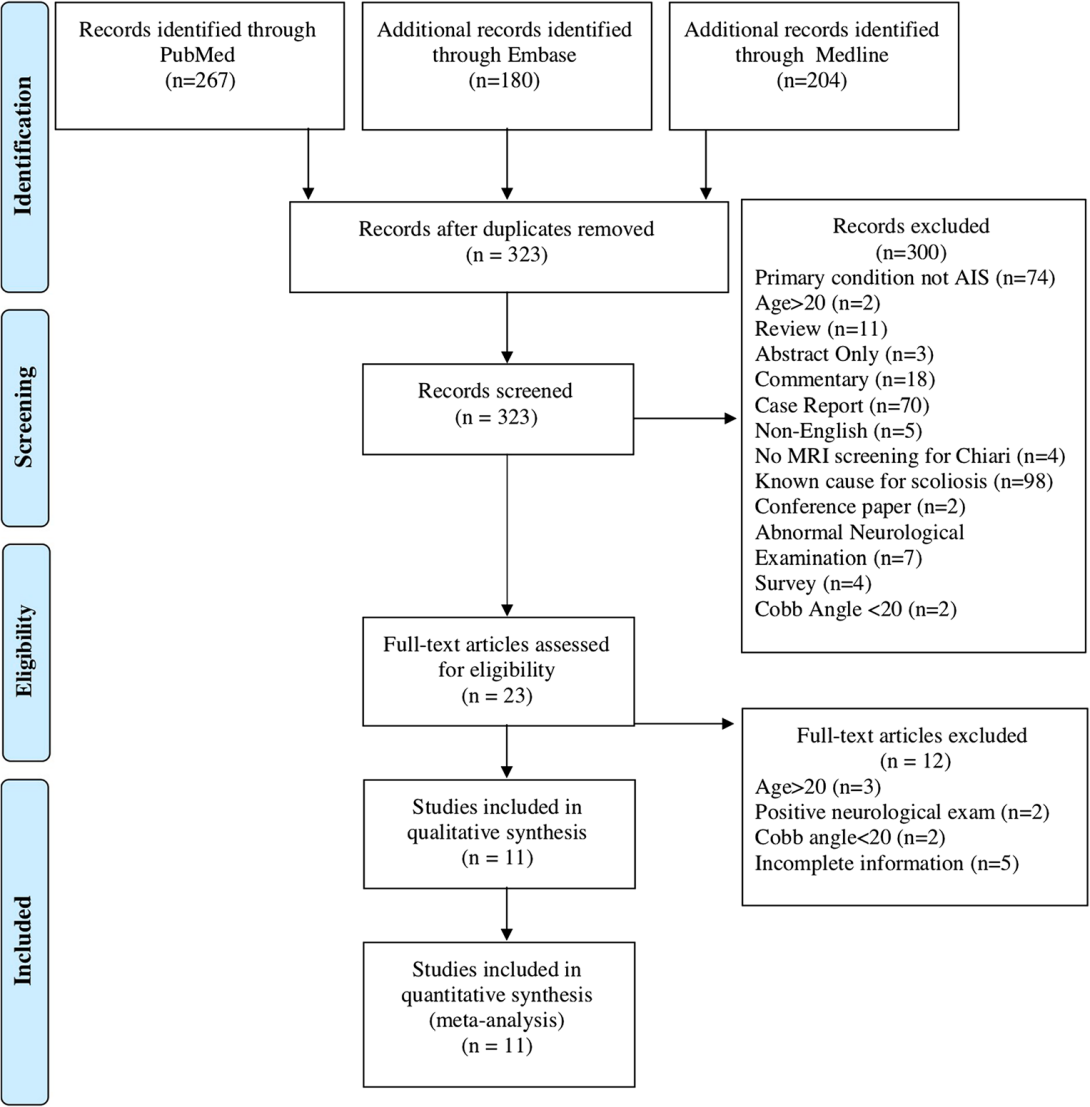
Eligibility criteria

Out of 323 studies initially found, 300 were excluded. After review of the full-text of the remaining 23 studies, 11 were found to meet the eligibility criteria (Fig. 1). Table 1 outlines the main findings. Out of 11 identified studies including 3372 paediatric patients with scoliosis and normal neurological examination, 495 (14.7%) were found to have a neuro-axial disease on full spine MRI. Two hundred eighty-one (8.3%) and 282 (8.4%) patients were found to have CM and syringomyelia in isolation. The mean patient age at scoliosis diagnosis was 9.9 years. Out of five studies reporting curve direction, 58.3% were right curves. Out of 10 studies reporting mean Cobb angle, the average was 38.9 degrees. The spectrum of neuro-axial disease encountered in addition to CM and/or syringomyelia included diastematomyelia, paraspinal-inter-spinal tumours, tethered cord, brainstem tumours, diffuse dural ectasia, low-lying conus and fatty filum. Table 2 provides an overview of the various disorders encountered.

**Table 1:** Summary of findings

Study	Year	Region	No. patients	Mean age	Mean Cobb (range)	Neuro-axial abnormality detected (%)	Chiari malformation detected (%)	Syringomyelia detected (%)
Zhang et al. [17]	2016	China	504	7.3	30.4 (20–64)	94 (18.7)	61 (12.1)	32 (6.3)
Strahle et al. [5]	2015	USA	1740	9.5	30.77 (NA)	323 (18.6)	186 (10.7)	209 (12.0)
Martin et al. [18]	2014	USA	43	1.3	35.6 (20–69)	7 (16.2)	2 (4.7)	3 (7)
Koc et al. [2]	2012	UK	72	3.7	46.6 (10–118)	8 (11.1)	6 (8.3)	7 (9.7)
Ozturk et al. [19]	2010	Turkey	249	14.3	55.6 (45–80)	20 (8)	5 (2)	18 (7.2)
Pahys et al. [20]	2009	USA	54	1.2	49.0 (20–109)	7 (13.0)	2 (3.7)	2 (3.7)
Inoue et al. [21]	2005	Japan	204	11.4	62.8 (NA)	44 (18)	35 (17.2)	24 (11.8)
Hausmann et al. [22]	2003	Switzerland	100	15.2	56 (NA)	3 (3)	1 (1.0)	2 (2.0)
Do et al. [23]	2001	USA	327	13.0	57 (40–98)	7 (2.2)	4 (1.2)	2 (0.6)
Gupta et al. [24]	1998	USA	34	8.9	–	6 (17.6)	1 (2.9)	2 (5.9)
Maiocco et al. [25]	1997	USA	45	15.4	56 (NA)	2 (4.4)	1 (2.4)	2 (4.4)
Mean weighted total		–	3372	9.9	38.9 (20–118)	521 (15.3)	304 (9.0)	303 (9.0)

**Table 2:** Scoliosis curve outcome for patients who underwent foramen magnum decompression

Author	Year	Study type	Population	Outcome
Eule [2]	2002	Retrospective	• 19 patients with scoliosis (12 M, 13 F) undergoing decompression ± reduction of syrinx • Age 19 months to 16.5 years (mean age 8.7 years) • 11 patients also had spinal fusion (57.9%) • 8 patients had no spinal fusion (42.1%)	• Younger age and decompression associated with better outcomes • Out of 8 patients without fusion, scoliosis progressed in 37.5%, stabilised in 12.5% and improved in 50%. Mean age of patients who progressed was 14.5 years whereas 6 years in those whose Cobb angle improved.
Brockmeyer [6]	2003	Retrospective	• 21 patients under 16 years of age with CMT1, scoliosis and no fusion during follow-up period after suboccipital decompression	• Scoliosis curve stabilisation or improvement in 62% of patients and worsening in 38%. 91% improvement or stabilised in under 10 years of age.
Ozerdemoglu [8]	2003	Retrospective	• 12 patients (group I) with scoliosis and syringomyelia but not congenital scoliosis or myelomeningocoele who underwent decompression	• 58.3% of patients had scoliosis curve improvement, 25% of patients had worsening and 16.6% of patients had no change. The greatest improvement was seen in children less than 10 years of age.
Tubbs [12]	2011	Retrospective	• 90 paediatric patients with CMT1 and scoliosis (82% of these had syringomyelia) and whom 44% also underwent spinal fusion	• Cobb angle > 40° was less likely to improve with posterior fossa decompression even when there was a decrease in the size of the syrinx.
Krieger [10]	2011	Retrospective	• 79 paediatric patients with scoliosis and CM-1 • 30 patients had scoliosis angle 25°–80°	• Of 30 patients who underwent decompression for scoliosis • 30% of patients had curve improvement (2 patients had back bracing prior to decompression) • 70% of patients had curve progression (36% of these required further instrumentation and fusion surgery)

## Discussion

This is the first review to assess the utility of full spine MRI for paediatric patients with presumed idiopathic scoliosis and normal neurological examination. It is an important study given the recent interest in the association between scoliosis and neuro-axial disease, in particular CM and/or syringomyelia [5]. CM is hypothesised to cause asymmetric compression of the cervico-medullary junction by the cerebellar tonsils, which predisposes to irregular contraction of the deep spine muscles, resulting in scoliosis even in the absence of syrinx [6, 7, 12, 13]. Traditional treatment for idiopathic scoliosis includes back bracing and invasive orthopaedic surgical intervention. Fusion procedures in particular are invasive and not without risk of complication. It is our opinion that in the presence of a neurological driver, traditional treatments may have limited effect. It is thus important to know whether patients may have such disease that is amendable to neurosurgical intervention. It is even possible that such intervention may improve outcome independent of traditional intervention.

In this review, 14.7% of patients with presumed idiopathic scoliosis and normal neurological examination demonstrate a neuro-axial disease on full spine MRI. This is despite a US report stating that traditional non-radiological testing is sufficient to diagnose adolescent idiopathic scoliosis [14]. This suggests that one in seven patients will have a potentially treatable neurological cause for (or contributory to) their scoliosis. Given the invasive nature of instrumentation and fusion procedures, these patients may benefit from such detection if prompt correction of neuro-axial disease can improve scoliosis outcome. Spinal surgery for adolescent idiopathic scoliosis has a 2.6% rate of perioperative major complications and 4.1% of major complications at two or more years post surgery [15]. This is not insignificant, particularly given the lifetime effect on younger patients. There are currently no prospective studies or RCTs that have investigated the treatment effect of neuro-axial disease with and without instrumentation of spinal fusion procedures.

Table 3 demonstrates studies that suggest scoliosis curve improvement in patient that undergo decompression. Four out of the 5 identified studies demonstrated a curve improvement of between 30 and 50% of patients [3, 6, 8, 10]. Several studies suggest that FMD prior to the age of 10 is most likely to improve or stabilise the scoliosis curve [3, 6, 8, 16]. Therefore, there is a clear window in which screening by MRI is both safe and enables an effective therapeutic intervention; however, it should be remembered that sedation is not without risk in a paediatric population. While there are small studies with patient groups younger than 6 which report anecdotally good outcomes, the evidence base is not currently sufficiently strong enough to justify the known additional risk of sedation or anaesthesia in a screening programme for a currently unproven surgical advantage at a younger age [26]. We discuss age as an important consideration given skeletal maturity is eventually reached at an older age, but this must be weighed against the risk of sedation. It is also difficult to correlate the intervention with the result given it is unclear whether there would be progression without FMD.

**Table 3:** Summary of other neuro-axial disease

Study	Year	% Tonsillar ectopia	% Diastematomyelia	% Paraspinal tumours	% Tethered cord	% Brainstem tumours	% Diffuse dural ectasia	% Low-lying conus	% Fatty filum
Zhang [17]	2016	0	1.2	0.8	0.8	0	0	0	0
Strahle [5]	2015	0	0	0	0	0	0	0	0
Martin [18]	2014	0	0	0	2.3	0	0	0	4.7
Koc [2]	2012	0	0	0	0	0	0	0	0
Ozturk [19]	2010	0	0	0	0	0	0	0	0
Pahys [20]	2009	0	0	0	5.6	0	0	0	0
Inoue [21]	2005	3.9	0	0	0	0	0	0.5	0
Hausmann [22]	2003	0	0	0	0	0	0	0	0
Do [23]	2001	0	0	0	0	0	0	0	0.3
Gupta [24]	1998	0	0	0	0	2.9	2.9	5.9	0
Maiocco [25]	1997	0	0	0.1	0	0	0	0	0
Mean weighted total		0.2	0.2	0.1	0.2	0.02	0.03	0.09	0.09

[27] guidelines for a screening test require that “there should be a simple, safe, precise and validated screening test” [27]. MRI is a simple, precise and validated test for detecting neuro-axial disease. Furthermore, the MRI itself without anaesthesia or contrast has less risk than the associated car drive to the hospital [28]. However, in a younger paediatric population, there are the considerable additional risks of general anaesthesia and airway support to consider. There are several studies which highlight the adverse effects of sedation in paediatric populations [29–31]. Heyer et al. [32] indicated that only 9% of children aged 4 and 2% of children over the age of 4 required being sedated for a brain MRI, while the UK experience is that sedation is unnecessary above the age of about 5 or 6 with adequate audio-visual distractions. It is promising that the mean patient age from all identified studies is 9.9 years, but consideration must be taken for younger children.

This study suffers from several limitations. The heterogeneity of the literature is not uncommon for this type of study. This is further challenged by the relative paucity of prospective literature. It remains a topic that is still relatively unexplored. Also, the increased utilisation and thus cost of MRI scanning will have to be absorbed by already financially stretched healthcare systems worldwide. Furthermore, the optimal age of benefit from FMD (< 10) also poses the increased risk of an anaesthetic for screening purposes. However, this study does have several strengths. Both retrospective and prospective studies were included, and given that it would be extremely difficult to undertake a randomised clinical trial in this area, this is the best quality of evidence available to date. Also, this study forms the foundation on which future national and international multicentre studies can build upon in a quest to more accurately determine the efficacy and feasibility of introducing an imaging-based screening program for paediatric scoliosis.

## Conclusion

Up to one in seven paediatric patients with scoliosis and normal neurological examination will demonstrate neuro-axial disease on MRI imaging of the spine. Given young and earlier age of decompression is associated with improvement in curve angle, it seems important that MRI screening be considered in all patients regardless of neurological examination findings. There is a potentially long-term benefit for these patients. Multi-institutional prospective studies are encouraged to further investigate effect on curve angle.

## Abbreviations

CM: Chiari Malformation
FMD: Foramen magnum decompression
MRI: Magnetic resonance imaging
NAD: Neuro-axial disease
RCT: Randomised control trial
